# The Impact of COVID-19 on Preschool-Aged Children’s Movement Behaviors in Hong Kong: A Longitudinal Analysis of Accelerometer-Measured Data

**DOI:** 10.3390/ijerph182211907

**Published:** 2021-11-12

**Authors:** Johan Y. Y. Ng, Qing He, Kar Hau Chong, Anthony D. Okely, Cecilia H. S. Chan, Amy S. Ha

**Affiliations:** 1 The Department of Sports Science and Physical Education, Kwok Sports Building, The Chinese University of Hong Kong, Shatin, Hong Kong, China; yyng@cuhk.edu.hk (J.Y.Y.N.); qinghe@cuhk.edu.hk (Q.H.); ceciliachanhs@cuhk.edu.hk (C.H.S.C.); 2 Early Start, Faculty of Arts, Social Sciences and Humanities, University of Wollongong, Wollongong, NSW 2522, Australia; khchong@uow.edu.au (K.H.C.); tokely@uow.edu.au (A.D.O.)

**Keywords:** moderate-to-vigorous physical activity, sleep, screen time, accelerometry

## Abstract

During the COVID-19 pandemic, many preschool-aged children were forced to remain indoors due to social distancing measures and school closures. In this study, we examined how children’s movement behaviors (sedentary behaviors, physical activity, and sleep) were affected by the pandemic. Children’s (*N* = 25, age = 4.4 years, *SD* = 0.3) movement behaviors were measured before and after the COVID outbreak, respectively. Data collected using accelerometers were analyzed using compositional data analyses. A significant change in the overall time-use composition (*F* = 5.89, *p* = 0.002) was found. Results suggested that children spent more time sleeping (8% increase) and in moderate-to-vigorous physical activity (16% increase), with less time spent in sedentary behaviors (9% decrease). However, parent reports suggested that children were less active and had more screen time. In conclusion, the current evidence suggests that children’s physical activity is not negatively impacted by the pandemic. However, the continuous surveillance of movement behaviors of young children during the pandemic is needed.

## 1. Introduction

The COVID-19 pandemic has brought enormous changes to our daily lives. During the height of the restrictions when preschools were closed, many young children, especially those living in urban areas, were limited to home-based indoor activities amid enforced social distancing measures. Such restrictions will likely have negative impacts on the physical activity (PA) and sedentary behaviors (SB) of preschool-aged children [1], and in turn affect their health and well-being. In 2019, the World Health Organization (WHO) [2] released PA recommendations for children under 5 years of age. In particular, the WHO recommended children aged 3 to 4 to accrue 180 min of PA per day, in which at least 60 min should be of moderate to vigorous intensity. Children are recommended to refrain from having more than 1 h of sedentary screen time, and to have 10 to 13 h of good quality sleep. Studies to date reporting the changes in children’s PA, SB, and sleep during COVID-19 have used parental report, which is prone to bias and inaccuracies [3]. To our knowledge, no study has examined the COVID-19-induced changes using a device-based measure of movement behaviors—such as accelerometry, which is a well-established, objective measure for PA. The purpose of this study was to examine the potential impact of the COVID-19 pandemic on young children’s 24-h movement behaviors in Hong Kong. We hypothesized that young children would spend less time in PA at various intensities after the onset of the COVID-19 pandemic relative to the time before social distancing measures were enforced.

## 2. Materials and Methods

### 2.1. Participants and Procedures

**Figure 1:**
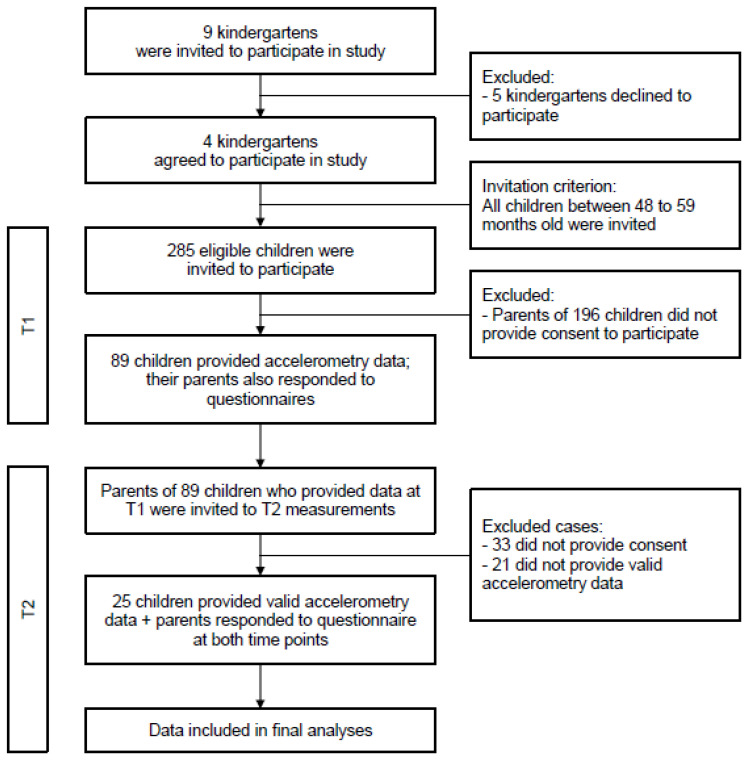
Flow diagram showing how the dataset used for analyses was derived.

The 24-h movement behaviors of children from four Hong Kong preschools were measured using accelerometers pre- (T1; April to December 2019) and during (T2; early June 2020) the COVID-19 outbreak. Children were recruited through preschools that responded to our invitation to take part in the International Study of Movement Behaviors in the Early Years (SUNRISE) [4]. The main objective of SUNRISE is to monitor the global proportion of 4-year-old children meeting the 24-h movement behavior guideline set out by the WHO [2]. Children were eligible if they were between 48 and 59 months old when the measurements were taken. A flow diagram depicting the details of enrollment and participation are shown in Figure 1.

At T1, all children were attending normal schooling, and accelerometers were administered to 89 children directly by researchers. Between late January and June 2020 (i.e., inclusive of T2), all preschools were suspended to prevent the spread of the virus. At the time when T2 data were collected, Hong Kong citizens were encouraged to not leave their homes for nonessential activities, and social gatherings of more than eight people were prohibited by law (at the peak, gatherings of more than two were disallowed). Parents of children who took part at T1 were contacted and invited to participate in the follow-up study, and the parents of 56 of them agreed to take part. Accelerometers assigned to these children were then sent to kindergartens and were then collected and returned by consented parents. After the removal of missing or invalid accelerometer data, the final sample consisted of 25 children (aged 4.4 ± 0.3 years at T1; 72% boys) who provided valid accelerometer data at both time points.

The protocol of the study was reviewed and approved by the Joint Chinese University of Hong Kong—New Territories East Cluster Clinical Research Ethics Committee (Ref: 2018.563). Parents of all participants provided written informed consent for their children to take part in the study, whereby they consented to providing accelerometer (their children’s) and questionnaire (parents’) data for analyses.

### 2.2. Measures

At both timepoints, ActiGraph wGT3X-BT accelerometers were placed on the children on Mondays, and the children were asked to wear the devices on their right hip, except when engaging in water-based activities, until the Friday of the same week. Devices were set to capture data at a sampling rate of 30 Hz, which was integrated into 15 s epochs for analyses. In this study, we compared data from children who provided at least one day of valid data (between Tuesday and Thursday) at both T1 and T2. Data were considered valid for the analysis if the child wore the accelerometer for sleep (determined through visual inspection of the acceleration graphs [4]) and at least eight hours during waking hours [4]. Non-wear time was flagged as 20 min or more of consecutive zeros [5] and was excluded from the analysis of wake time data. Daily time spent in each movement behavior (sleep, SB, light-intensity physical activity [LPA], and moderate-to-vigorous intensity physical activity [MVPA]) were calculated. Average bedtimes, waking times, and sleep durations were determined using visual inspection rules, as previously reported [6,7]. Time spent SB, in LPA, and in MVPA were calculated using Pate et al.’s [8,9] cut points. 

Parents also reported sleep timings (i.e., bedtime, wake up time, and daytime napping), sedentary screen time, total PA, MVPA, and the time children spent outdoors at both time points using questionnaires. Parents were invited to complete the questionnaires after their children returned the accelerometers, so that their response should correspond to the same period in which the devices were worn. Specifically, the sleep duration was calculated based on the parent-reported bedtime and wake-up times of children, plus their nap durations. For the PA outcomes, parents responded to a series of questions, with a stem of “On a 24 h period in the past week, how much time did your child spend on”, in relation to “any electronic screen device such as a smart phone, tablet, video game, or watch television of movies, videos on the internet while they were sitting or lying down” (sedentary screen time), “a variety of physical activities, spread throughout the day” (total PA), and “energetic play that causes him/her to ‘huff and puff’ and increases his/her heart rate” (MVPA). Times children spent outdoors were measured using two questions in the format of: “On a typical weekday (weekend day), how much time does your child spend outside?” All parent questionnaire items were translated and administered in Chinese. For this paper, these data were used to examine the potential differences in device-measured and parent-reported movement behaviors. 

### 2.3. Data Analyses

The movement behaviors measured (i.e., sleep, SB, LPA, and MVPA) are mutually exclusive and exhaustive components within a 24-h period. Therefore, they should be analyzed and interpreted in relation to each other using a compositional data analysis approach [10,11]. Specifically, a four-part movement behavior composition (SLEEP–SB–LPA–MVPA) was created for each child at each time point by transforming the accelerometer-measured movement behavior data into a set of three isometric log-ratio (*ilrs*) coordinates. The *ilrs* contains all the relative information about the movement behavior composition. Repeated measures MANOVA was then employed to assess if the set of *ilrs* changed from T1 to T2. If significant, to explore what behaviors are driving the change in the composition, the log-ratio difference of the compositional means between the time points (*ln*[T2/T1]) and its 95% bootstrap confidence interval for each behavior were derived and plotted for interpretation [12,13]. A change was considered significant if its 95% bootstrap confidence interval did not encompass zero. Changes in the WHO guideline adherence rates from T1 to T2 were examined using the McNemar’s exact test. Comparisons between the parent-reports at both time points were conducted using the Wilcoxon signed-rank tests. An alpha level of 0.05 was used.

## 3. Results

To ensure that the children who took part in the follow-up study had similar characteristics to those who did not, we first examined whether there were differences between these two samples. At T1, the sample included in the final analyses had a shorter mean sleep duration than those who did not take part in the follow-up (575 ± 40 min/day vs. 602 ± 67 min/day; *p* = 0.021). By contrast, we found no differences in terms of their accelerometer-measured SB, LPA, and MVPA. Further, no significant differences were observed in the parent-reported durations for total PA and sedentary screen time between the two samples. Based on these results, we deemed that there was insufficient evidence to suggest that the two samples had different characteristics, and thus no indication of potential selection bias.

**Figure 2:**
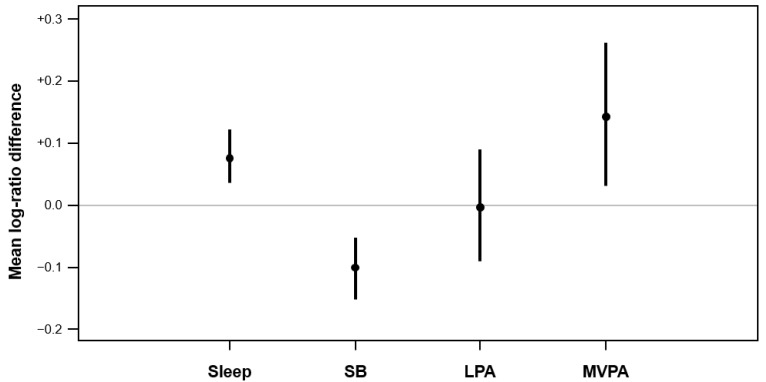
Bootstrapped log-ratio differences of compositional means of movement behaviors between T1 and T2. The log-ratio difference was calculated by using the compositional means of behavior at T2 as the numerator and the compositional means at T1 as the denominator (T2/T1). A positive value of the log-ratio difference (i.e., above zero) indicates that children spent more time in that behavior at T2 than T1. The change within each movement behavior component is considered significant if its 95% bootstrap confidence interval does not cross the zero line. *SB* = sedentary behavior; *LPA* = light physical activity; *MVPA* = moderate-to-vigorous physical activity.

Descriptive statistics of the measured outcomes are presented in Table 1. A significant change in the overall time-use composition (*F* = 5.89, *p* = 0.002) was found. There were significant differences in the children’s time spent in sleep, SB, and MVPA, but not in LPA (Figure 2). Specifically, the children’s SB decreased by 9% from T1 to T2. By contrast, their time spent in sleep and in MVPA increased by 8% and 16%, respectively. The increase in sleep duration was due to participants averaging later bedtimes (by 33 min; 95% CI (0:05, 1:02)) and later wake-up times (by 78 min; 95% CI (0:52, 1:44)) at T2. There was no significant change in the proportion of children who met the WHO guidelines for sleep (T1: 16% (*n* = 4) vs. T2: 40% (*n* = 10); *p =* 0.110), total PA (light to vigorous PA; 76% (*n* = 19) vs. 92% (*n* = 23); *p* = 1.00), and MVPA (68% (*n* = 17) vs. 72% (*n* = 18); *p* = 0.125). In terms of questionnaire-based data, parents reported that children spent more time in sedentary screen time (95% CI (39.2, 97.6)), which resulted in a smaller proportion meeting the WHO guideline (*p* = 0.012). Based on the parent-reports, the children spent less time outdoors during weekdays (95% CI (−101.5, −42.1)) and weekends (95% CI (−170.7, −49.3)). Due to a small cell size (*n* < 5) in the analyses for sleep data, the results of these analyses should be interpreted with caution.

### Sensitivity Analyses

At T2, four children (16%; none at T1) only had valid accelerometer data on one day. To ensure that our results were not affected by the shorter monitoring periods of these children, a sensitivity analysis was conducted by repeating our analyses after excluding those who only had one day of valid accelerometer data at T1. Similar to our main analyses, a significant change in time-use composition was found (*F* = 5.31, *p* = 0.004). 

## 4. Discussion

Contrary to other findings based on parent or children self-reports [14,15,16,17,18,19], the accelerometer-based results from our study sample show that children’s PA did not decrease as a result of the pandemic. We found that the MVPA of children increased from T1 to T2, with a difference of 15 min per day. We also found no changes in LPA and a significant reduction in time spent in SB. This suggests that additional time spent indoors does not directly imply a reduced PA and increased SB. In fact, one might infer that, compared with being at home, children’s PA might be more compromised when they are attending school [20], which might be a result of extended time students spend sitting (i.e., SB) when receiving instruction in preschools in Hong Kong. This highlights the need to incorporate more active learning experiences for preschool children at kindergartens, which has also been shown to improve learning outcomes [21]. Apart from accruing PA, preschool education is tasked with developing children’s motor skills, which may affect children’s future PA participation. Researchers should therefore examine changes in children’s development in motor domains as a result of the lack of formal face-to-face instruction.

We found increases in children’s sleep duration. This appeared to be a result of later bedtimes and wake-up times. Earlier bedtimes are considered important for sleep promotion [22]. Despite there being a temporary increase in total sleep duration, deferred bedtime may still have negative implications to children’s health [23]. Further, children would likely have to return to usual wake-up time when normal schooling resumes, and if their bedtime remains unchanged, their total sleep duration may be reduced below pre-COVID-19 levels. 

The marked increases in parent-reported sedentary screen time were also noteworthy. Based on the parent-reports, children’s screen time increased by 69 min per day. This is worrisome, as sedentary screen time may be associated with sleep quality and other health consequences [24]. Nonetheless, many kindergartens have adopted one or more forms of screen-based instruction while children cannot return to school physically [25]. This may increase children’s overall sedentary screen time. Since young children’s screen time (both sedentary and active) is dependent on parents’ behaviors [26], they should be empowered to actively reduce children’s excessive sedentary screen viewing behaviors. As these results were based on parent-reports, they should be interpreted with caution.

Results of this study highlight the importance of including objectively measured outcomes in research in this age group. We found large discrepancies between the device-measured and the parent-reported outcomes in terms of changes in children’s movement behaviors. Parents reported that children spent less time sleeping, while accelerometer-based results suggested the opposite. Similarly, the parents reported a 42% reduction in children’s total PA, yet these findings were not supported by the device-measured outcomes. Such differences are understandable, especially for working parents, as children might not have woken up by the time they left for work. They are also unable to closely follow the actual behaviors of at-home children during working hours. Nonetheless, this illustrates the necessity of including device-based movement behavior outcomes in research pertaining preschool-aged children. Relying solely on parent-reported data may result in questionable results. 

To the authors’ knowledge, this is the first study that has examined the effects of the COVID-19 pandemic on preschool-aged children’s physical activity behaviors using objective tools. Further, this may also be the first paper that has applied compositional data analyses to examine the concurrent changes in time allocation between various types of movement behaviors, across various times, during the pandemic. These are the strengths of this study. By contrast, a relatively modest sample size may be seen as a potential limitation to the study. This was partially due to parents being reluctant to participate in T2 data collection during the pandemic for hygienic reasons. The seemingly small sample may harm the statistical power of the study. However, to the knowledge of the authors, there are currently no methods to calculate the power for compositional data analytic approaches. Nonetheless, we did find significant findings in our analyses, and hence the limitations in statistical power should not be of great concern. To avoid further reduction in sample sizes, we have also considered the accelerometry data to be valid if participants had one day of valid data at both time points. This criterion may seem relatively loose compared to other existing studies. However, we applied a slightly more restrictive valid day criteria in this study, whereby wearing the device to sleep was necessary. We would argue that young children’s movement behaviors during weekdays, especially when stay-at-home policies are enforced, are relatively stable. Hence, the data from only one day would still be representative. However, additional evidence is required to examine the reliability of movement behaviors under these challenging circumstances, and hence the results from our study should be interpreted with some caution.

Due to the nature of the study, we were unable to examine the potential confounding effect of children’s maturation on their 24-h movement behaviors. Specifically, researchers showed that the time children in this age range spent in LPA and MVPA may be on an increasing trend [27,28]. Yet in these studies, the SB of children remained the same. Hence, another interpretation of our results was that the observed changes in movement behaviors were merely a reflection of usual maturation. At present, unfortunately, there is insufficient local data to enable a formal comparison or investigation. Further studies are needed to establish the longitudinal changes in young children’s movement behaviors.

## 5. Conclusions

Preliminary evidence suggests that young children’s accelerometer-measured PA behaviors were not negatively impacted by the COVID-19 pandemic, with the exception of the increases in sedentary screen time. Nonetheless, we should continue to promote healthy movement behavior patterns in children during these difficult times [29]. The potential impacts on children’s physical and mental health [30] should be closely monitored, as any ill effects at this important development stage of students may not surface immediately but could have long-lasting effects until the later stages of children’s lives.

## Figures and Tables

**Table 1:** Descriptive statistics of measured variables at pre- (T1) and during COVID-19 (T2).

Variable	T1	T2
*Accelerometry-measured variables—Compositional means (min/day) ^a^*		
Sleep duration	580	627
Sedentary behavior	663	601
Light physical activity	107	107
MVPA	90	105
*Sleep schedule*		
Bedtime	22:50 (0:57)	23:24 (1:19)
Wake-up time	08:00 (0:34)	09:19 (1:20)
*Parent-reported measures*		
Sleep duration	688 ± 70	652 ± 64
Sedentary screen time	78 ± 54	147 ± 95
Total physical activity	143 ± 71	82 ± 73
MVPA	36 ± 35	23 ± 26
Time spent outdoor (weekdays)	127 ± 92	55 ± 60
Time spent outdoor (weekends)	237 ± 144	127 ± 131
*Adherence to WHO guidelines (n and percentage)*		
Sleep	4 (16%)	10 (40%)
Sedentary screen time	14 (56%)	5 (20%)
Total physical activity	19 (76%)	23 (92%)
MVPA	17 (68%)	18 (72%)

Note. Data presented as mean (standard deviations), unless otherwise indicated. MVPA = moderate-to-vigorous physical activity. ^a^ Geometric mean of minutes spent in each movement behavior component that has been adjusted to 1440 min (24 h).

## Data Availability

Data of this study could be provided upon reasonable request to the corresponding author.
